# Prevalence and predictors of vitamin D-deficiency in frail older hospitalized patients

**DOI:** 10.1186/s12877-018-0919-8

**Published:** 2018-09-20

**Authors:** Simon Friedrich Boettger, Bjoern Angersbach, Christiane Nicola Klimek, Ana Lemos Monteiro Wanderley, Azim Shaibekov, Lars Sieske, Baigang Wang, Matthias Zuchowski, Rainer Wirth, Maryam Pourhassan

**Affiliations:** Department of Geriatric Medicine, Marien Hospital Herne, University Hospital, Ruhr-University Bochum, Hölkeskampring 40, D-44625 Herne, Germany

**Keywords:** Vitamin D deficiency, Geriatric, Older, Frail, Falls

## Abstract

**Background:**

Vitamin D deficiency is known to be highly prevalent in older persons. However, the prevalence in the subgroup of frail older hospitalized patients is not clear. We sought to investigate the prevalence and predictors of vitamin D deficiency in frail older hospitalized patients.

**Methods:**

217 consecutively geriatric hospitalized patients with routine measurements of 25-hydroxyvitamin D [25 (OH)D] at hospital admission were analyzed retrospectively, including information of previous vitamin D supplementation and the geriatric assessment. Serum 25 (OH)D concentrations < 20 ng/ml and between 20 and 29.99 ng/ml were classified as deficient and insufficient, respectively, whereas concentrations ≥30 ng/ml were considered as desirable. A stepwise binary logistic regression model was performed to assess the simultaneous effects of age, gender and geriatric assessments on the prevalence of low vitamin D concentration.

**Results:**

Mean age of the cohort was 81.6 ± 8.0 years (70.0% females). Mean serum 25(OH)D was 12.7 ± 12.9 ng/ml. Of 167 (77%) subjects without known previous vitamin D supplementation, only 21 (12.6%) had serum concentrations ≥20 ng/ml and only 8 (4.2%) had desirable serum concentrations ≥30 ng/ml. In total population, 146 (87.4%) participants were vitamin D deficient. Despite vitamin D supplementation, 22 of 50 participants (44.0%) were vitamin D deficient and only 19 (38.0%) had desirable concentrations of ≥30 ng/ml. In a stepwise logistic regression analysis, only previous intake of vitamin D supplementation and high Geriatric Depression Scale score (GDS-15) were significantly associated with vitamin D deficiency.

**Conclusions:**

In the group of frail older hospitalized patients without previous vitamin D supplementation, the prevalence of inadequate vitamin D concentrations is extremely high. Therefore, usefulness of the routine measurement of vitamin D status before initiating of supplementation appears to be questionable in this patient group.

## Background

Vitamin D supplementation has been shown to reduce the risk of falls and fractures in older subjects with vitamin D deficiency [1]. However, several randomized controlled trials indicated that high dose vitamin D supplementation is associated with an increased risk of falls and fractures in older individuals [2–4]. Besides the primary effects on intestinal calcium absorption, renal calcium excretion and bone mineralization, vitamin D has been linked to skeletal muscle strength and lower extremity function as a line of evidence to the risk of falls [5]. Recently, vitamin D has also been linked to cardiac function in a prospective randomized trial [6].

Small proportion of vitamin D is obtained from food [7]. Its main natural source is the production in the sunlight exposed skin. Thus, the vitamin D skin synthesis of older persons is compromised by many factors including deprivation of sunlight exposure due to decreased mobility and a physiologic atrophy and reduction of the synthesis performance of the aging skin [8]. In addition, malnutrition is very prevalent in older persons and an alteration of nutritional intake and avoidance of fish and dairy products may also contribute to lower vitamin D concentrations.

It is well known that vitamin D deficiency is very prevalent in the older population [9–11]. Nevertheless, there is some controversy about the optimal blood level of 25-hydroxyvitamin D [25 (OH)D]. The Institute of Medicine (IOM) suggests serum concentrations of 25 (OH)D above 20 ng/ml as vitamin D sufficiency in the general population [12]. However, the Endocrine Society recommends serum concentration of 25 (OH)D above 30 ng/ml as desirable whereas concentrations less than 20 ng/ml and concentrations between 20 and 30 ng/ml were considered as 25(OH)D deficiency and insufficiency, respectively [13, 14]. Although the different relationship of serum concentration of 25(OH)D to parathyroid hormone (PTH) levels in different age and sex groups implies various target ranges for different groups, good scientific evidence indicates that the prevalence of secondary hyperparathyroidism is lowest with 25(OH)D- concentration above 30 ng/ml [13]. Finally, most interventional studies reaching at least a level of 30 ng/ml have determined a fall risk reduction, whereas studies reaching mean concentration of 20 ng/ml did not [13]. Therefore, a target level of ≥30 ng/ml is regarded to be adequate for geriatric patients with high risk of falls and osteoporosis [14].

To our knowledge, the prevalence of vitamin D insufficiency and deficiency in frail older hospitalized patients is expected to be high but is not well studied and predictors are unknown. On the other hand, routine assessment of serum concentration of 25(OH)D status is very expensive, with costs of about 20 € per analysis. Accordingly, these resources could possibly be more effectively invested in a sufficient vitamin D supplementation, if the prevalence of vitamin D insufficiency is extremely high. Our hypothesis is that the prevalence of vitamin D insufficiency is extremely high among frail older hospitalized patients and immobility may be a predictor of vitamin D deficiency.

## Methods

This study was part of a bigger study of 679 older participants (mean age 82.1 ± 8.2 and mean weight 70.5 ± 17.6 kg; 457 females) [15]. In the current study, in order to measure the prevalence of vitamin D-deficiency and –insufficiency and their association with the results of the geriatric assessment, we retrospectively analyzed the routine measurements of serum 25(OH)D of 217 older patients which were consecutively hospitalized between 01.07.2016 and 31.10.2016 to a geriatric hospital ward. Serum 25(OH)D concentrations were obtained from the local laboratory data base. The measurements were performed at hospital admission with an electro-chemiluminescence immunoassay (ECLIA) on Cobas 8000 (e602), Roche, Mannheim, Germany. Serum 25(OH)D concentrations below 20 ng/ml were regarded as deficiency, concentrations between 20 and 29.99 ng/ml were classified as insufficiency and concentrations of 30 ng/ml and above were considered as desirable.

In addition, results of the routine geriatric assessment including Barthel-Index, Mini Mental State Examination (MMSE), the 15 item Geriatric Depression Scale (GDS-15), Mini Nutritional Assessment Short Form (MNA-SF) as well as previous intake of vitamin D supplementation were derived from the patients’ medical records. Briefly, the range of the German version of the Barthel-Index is 0–100 pts., with 100 pts. indicating independency in all activities of daily living [16, 17]. MNA-SF [18] was used for nutritional assessment and subjects were grouped as malnourished (0–7 points), at risk of malnutrition (8–11 points) and having normal nutritional status (12–14 points). Depression was investigated by the use of the GDS-15 [19] with the score < 6 as normal emotional status and a cognitive status was assessed using the MMSE [20] with the score ≥ 24 as normal cognitive. All assessments were performed soon after hospital admission.

### Statistical analysis

The statistical analysis was performed with SPSS statistical software (SPSS Statistics for Windows, IBM Corp, Version 23.0, Armonk, NY, USA). Continuous variables are reported by their means and standard deviations (SDs) for normally distributed variables and median values with interquartile ranges (IQR) were expressed for non-normaly distributed data. Categorical variables are expressed as absolute numbers and relative frequencies (%). A group comparison was performed using the *t*-test for continuous data with normal distribution, the Mann-Whitney *U* test for continuous variables with non-normal distribution and Pearson Chi-square test for categorical variables. A stepwise binary logistic regression model was performed to assess the simultaneous effects of age, gender and geriatric assessments (i.e. Barthel-Index, MMSE, GDS-15 and MNA-SF) on the prevalence of low vitamin D. A *P*-value of < 0.05 was considered as the limit of significance.

## Results

Baseline characteristics of study participants stratified by different serum 25(OH)D concentrations are presented in in Table 1. The mean age of the participants was 81.6 years and median Barthel-Index and MMSE were 40 pts. and 24 pts., respectively, representing a frail older population of hospital patients. In total population, mean serum 25(OH)D was 12.7 ng/ml. 168 (77.4%) patients had serum 25(OH)D concentrations below 20 ng/ml whereas, only 26 (12%) participants had desirable serum 25(OH)D concentrations above 30 ng/ml (Table 1).

**Table 1:** Characteristics of study participants stratified according to different serum 25(OH)D levels

	All	Deficiency (< 20 ng/ml)	Insufficiency (20–29.99 ng/ml)	Normal (≥ 30 ng/ml)	*P* value
Total, N (%)	217 (100)	168 (77.4)	23 (10.6)	26 (12.0)	
Female, N (%)	152 (70.0)	112 (73.7)	18 (11.8)	22 (14.5)	0.117
Male, N (%)	65 (30.0)	56 (86.2)	5 (7.7)	4 (6.2)	
Age, years	81.6 ± 8.0	81.4 ± 8.3	83.3 ± 5.8	81.7 ± 8.3	0.494
Vitamin 25(OH)D, ng/ml	12.7 ± 12.9	6.8 ± 5.2	24.0 ± 2.9	40.7 ± 9.3	0.001
25(OH)D supplementation +, N (%)	50 (23.0)	22 (44.0)	9 (18.0)	19 (38.0)	
25(OH)D supplementation -, N (%)	167 (77.0)	146 (87.4)	14 (8.4)	7 (4.2)	0.001
Barthel-Index, pts.	40 (30–50)	40 (25–55)	35 (25–45)	40 (35–55)	0.446
MMSE, pts.	24 (19–27)	23 (19–27)	25 (21–27)	24 (16–27)	0.472
GDS-15, pts.	2 (0–3)	3 (1–3)	1 (0–3)	2 (0–3)	0.052
MNA-SF, pts.	8 (6–10)	8 (6–10)	9 (7–10)	9 (5–10)	0.427

25(OH)D supplementation +, previous 25(OH) supplementation; 25(OH)D supplementation -, no previous 25(OH) supplementation; *MMSE* Mini-Mental-State-Examination, *GDS-15* 15-item geriatric depression scale, *MNA-SF* Mini-Nutritional-Assessment short form; Values are given as number (%), mean ± SD or median (interquartile range)

According to the patients’ medical records, there was evidence for previous intake of vitamin D supplementation in 50 patients in which 19 (38%) participants had sufficient concentrations above 30 ng/ml. However, doses and duration of vitamin D supplementation was unspecified in this group. In addition, in the group without previous vitamin D supplementation based on the patients’ medical records, 146 (87.4%) and 14 (8.4%) were vitamin D deficient and vitamin D insufficient respectively, whereas only 7 (4.2%) patients showed desirable concentrations of 30 ng/ml and above.

In a stepwise logistic regression analysis, the effects of age, gender and results of geriatric assessment (as independent variables) on the prevalence of low vitamin D were tested (Table 2). Only previous intake of vitamin D supplementation and high GDS-15 score were significantly associated with vitamin D deficiency.

**Table 2:** Binary logistic regression analysis of factors associated with serum 25(OH)D deficiency

	Beta Coefficient	SE	OR (95% CI)	*P* value
Age	−0.013	0.037	0.987 (0.918–1.061)	0.728
Gender	−1.150	0.738	0.317 (0.075–1.345)	0.119
25(OH)D suppl.+	−2.174	0.704	0.114 (0.029–0.453)	0.002
Barthel-Index	0.015	0.019	1.015 (0.978–1.052)	0.434
MMSE	−0.059	0.056	0.943 (0.844–1.053)	0.296
GDS-15	0.390	0.182	1.477 (1.034–2.110)	0.032
MNA-SF	−0.102	0.109	0.903 (0.730–1.117)	0.349

*25(OH)D suppl.+* Previous vitamin D supplementation, *MMSE* Mini-Mental-State-Examination, *GDS-15* 15-item geriatric depression scale, *MNA-SF* Mini-Nutritional-Assessment short form, *OR* Odds ratio, *SE* Standard error

## Discussion

The prevalence of vitamin D deficiency is generally considered to be high, even in the general population. In the National Health and Nutrition Examination Survey (NHANES), 41.6% of adult participants showed 25(OH)D concentrations < 20 ng/ml [21]. Due to decreased sunlight exposure and lower capacity of skin synthesis, the prevalence rates of vitamin D deficiency in older subjects could be expected to be even higher, especially in frail older hospitalized patients, as observed in our study cohort. In the current study, in the subgroup without recorded previous vitamin D supplementation, only 4.2% of participants showed desirable vitamin D concentrations. The prevalence data of our study cohort was comparable to three other existing studies in older hospitalized patients. In 412 orthogeriatric patients (mean age 80 years) with hip fracture in Singapore, vitamin D deficiency was present in 57.5%, vitamin D insufficiency in 34.5% and only 8% had desirable vitamin D concentrations > 30 ng/ml [11]. However, in that study only being housebound and Malay ethnicity were associated with vitamin D deficiency in a multivariate model. In another study of 123 Portuguese hospitalized patients in Internal Medicine (mean age 71 years), 92.7% presented with vitamin D deficiency and only 7.3% had concentrations > 20 ng/ml [22]. In addition, less than 5% of 332 hospital patients from Brussels (mean age 69 years) reached vitamin D concentrations > 30 ng/ml [9]. Moreover, it is worth mentioning that serum concentrations of vitamin D differ considerably during the seasons, reaching to the highest level in late summer and to the lowest level in late winter [23]. In a study of 4149 participants (aged 45–75 years) [24], the highest prevalence of vitamin D deficiency was observed in February/March (92%) whereas the lowest prevalence was seen in June/July (71%). However, our recent study in older hospitalized patients [15] demonstrated neither monthly nor seasonal variations in vitamin 25(OH)D concentrations; indicating that sunlight dependent skin synthesis is unlikely to contribute to vitamin D status in these patients. Therefore, supplementation seems to be necessary to maintain desirable vitamin D concentrations among this population throughout the year.

Due to high prevalence of vitamin D deficiency and insufficiency observed in our study and previous studies in hospital patients, it appears abdicable to measure vitamin D concentrations before initiation of supplementation in frail older hospitalized patients, because the pretest-probability for low serum-levels is 96%. Even predictive models which have been applied in community dwelling older subjects [25] appear impractical in our study population, as simply the hospital setting in frail older patients with the absence of previous supplementation reveals a 96% risk of having no adequate vitamin D level. In the present study, due to the very heterogenic study cohort, neither parameters of mobility nor nutritional assessment were linked to serum 25(OH)D concentrations. In addition, many of the patients suffered from acute disease in order that the Barthel-Index obtained on admission does not reflect their previous mobility. Furthermore, the significant association of vitamin D deficiency with a higher score of the geriatric depression scale is not surprising, since vitamin D deficiency has been linked to depression in many cross-sectional studies [26, 27]. However, there is a lack of evidence for its causality in interventional trials [27]. In a study of 1282 older patients aged 65 to 95 years, Hoogendijk et al. [28] indicated that serum concentrations of 25 (OH) D were 14% lower in both groups with minor and major depression. Eyles et al. [29] reviewed the possible association between low levels of vitamin D and neuropsychiatric diseases and demonstrated that vitamin D insufficiency or deficiency may impact on various neurotransmitter targets (namely, dopamine, noradrenaline and adrenaline). In addition, in some cross-sectional studies [30, 31], no relationship was observed between depression and vitamin D deficiency after considering the lifestyle factors such as time spent outside and the degree of sun exposure.

In general, testing for a condition which is prevalent in 96% of patients appears to be ineffective, especially if the condition may be mastered by supplementation at low costs and without side effects. However, there is controversy regarding the dose of vitamin D_3_ supplementation in achieving optimal serum 25(OH)D concentrations. Vitamin D_2_ in the dose range of 200,000 to 300,000 IU was effective in increasing mean 25(OH)D concentrations at 12 weeks in older frail patients who had low 25(OH)D concentrations at baseline [32] or a long-term efficacy of a 500,000 IU dose vitamin D_3_ were similarly effective in stabilizing the 25(OH)D concentration above 30 ng/mL at 12 weeks in older women [3]. In addition, in a recent study on vitamin D supplementation in older patients with low-trauma falls [5], the serum 25(OH)D concentrations increased by 12.7 ng/ml at 6 months with a dose of 24,000 IU per month. Therefore, we would not expect desirable serum concentrations before 6 months of supplementation at a daily dose of 800 to 1000 IU in frail older hospitalized patients without previous supplementation. We would therefore recommend initiating vitamin D supplementation in this population without testing the serum concentrations and assessing serum levels after 6 months of supplementation. When the serum 25(OH)D concentrations do not rise above 30 ng/ml, supplementation should be continued with an increased dose.

Previous studies have demonstrated the statistically significant decrease in the risk of falls and fractures associated with administration of vitamin D among older individuals [2, 33]. In a randomized controlled trial comprising 625 older people, Flicker et al. [33] revealed that administration of ergocalciferol 10,000 IU once weekly and then 1000 IU daily decreased the risk of fractures and falls. However, detrimental impact of high dose of vitamin D supplementation was observed in a several studies [3, 4]. Annual oral administration of 500,000 IU cholecalciferol in 2265 community-dwelling women (aged≥70 years) was followed by an increased risk of falls and fractures [3]. Consequently we do not recommend a high dose treatment.

Some limitations of the present study need to be mentioned. First, it is the retrospective methodology and we did not assess some factors such as BMI or dietary habits of the patients. Second, we have no information about the duration, doses and compliance of previous vitamin D supplementation. As the data were derived from the patients’ medical records, it cannot be excluded that some subjects had previous vitamin D supplementation without respective information in the record. Third, frailty was not defined by exact frailty criteria, but the results of the geriatric assessment and our experience with these patients indicate an entirely frail cohort. Finally, the relatively small sample size of total population may have limited the value of our results.

## Conclusion

Almost 96% of patients had serum 25(OH)D concentrations below desirable concentrations of ≥30 ng/ml. Since the pretest-probability of insufficient vitamin D levels in the group of frail older hospitalized patients is 96%, the measurement of serum vitamin D concentrations appears to be abdicable before the initiation of supplementation. Therefore, testing of serum 25(OH)D should be used for monitoring of supplementation rather than giving the indication for the initiation of supplementation in frail older hospitalized patients.

## Abbreviation

25 (OH)D: 25-hydroxyvitamin D
GDS-15: Geriatric Depression Scale
IQR: Interquartile ranges
MMSE: Mini Mental State Examination
MNA-SF: Mini Nutritional Assessment Short Form
PTH: Parathyroid hormone
SDs: Standard deviations
